# Microwave ablation: initial experience in Brazil

**DOI:** 10.1590/0100-3984.2019.0096

**Published:** 2020

**Authors:** Marcello Silveira Rovella, Lucas Fiore, Alfredo Enzo Allegro Filho, Guilherme Lopes Pinheiro Martins, Marcos Roberto Menezes

**Affiliations:** 1 Radiology Department, Instituto do Câncer do Estado de São Paulo (Icesp) - Radiologia, São Paulo, SP, Brazil.

## INTRODUCTION

Interventional radiology is a field of medicine that has grown significantly in recent decades. Technological advances, together with increased experience on the part of interventional radiologists as well as their interaction with oncologists and professionals in other medical specialties, have provided an important place for interventional radiology within the therapeutic arsenal for the treatment of neoplastic diseases^(1)^. Within that context, there are thermal ablation techniques such as radiofrequency ablation, cryoablation, and microwave ablation, which are minimally invasive means of treatment involving the insertion of specific needles or image-guided antennae, in order to treat focal lesions.

Microwave ablation involves the use of a generator that produces an electromagnetic field, transmitted by an antenna, which causes agitation and friction among water molecules, thus increasing the temperature of the tissue to 60-150°C, resulting in coagulative necrosis^(2)^. Microwave ablation has the following advantages over radiofrequency ablation^(3,4)^: producing higher temperatures, with the ability to ablate larger areas in less time; having less heat sink interference; and being unaffected by the high impedance of certain tissues. This therapeutic modality has been in clinical use since the 1990s^(5,6)^, with exponential increases in its use at various sites, mainly the liver, lung, kidney, and bone. However, its use has been authorized in Brazil only since the end of 2018.

The first two cases in which microwave ablation was performed in Brazil, one a case of lung injury and the other a case of liver injury, were both treated in May 2019 at the São Paulo State Cancer Institute.

## PROCEDURES

### Case 1 - Lung (metastasis from a sarcoma)

A 53-year-old female patient who had undergone resection of a pleomorphic sarcoma of the thigh in November 2015 presented with a single metastasis, measuring 1.8 cm, in the right lung and underwent radiofrequency ablation in January 2018. During the oncological follow-up, she presented a new single metastasis, measuring 1.0 cm, in the left lung in April 2019, and the decision was made to treat the lesion with microwave ablation.

**Figure 1:**
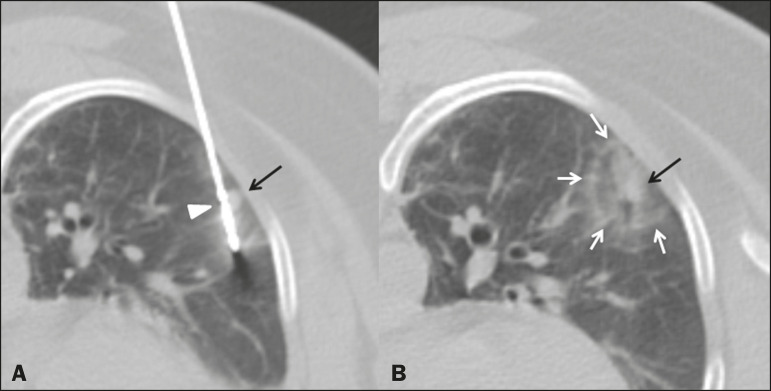
**A:** Chest computed tomography showing insertion of the microwave antenna (arrowhead) into the secondary pulmonary nodule (arrow). **B:** Follow-up computed tomography scan showing that the ablated area encompassed the entirety of the treated nodule (black arrow), with satisfactory margins (white arrows).

The procedure was performed under general anesthesia with a microwave tissue ablation system (Solero MTA; AngioDynamics, Latham, NY, USA) and was guided by computed tomography with a 40-channel scanner (Brilliance; Philips Medical Systems, Eindhoven, The Netherlands). The microwave antenna was inserted percutaneously into the lung lesion (Figure 1A), and microwave power was applied in two cycles: 2 min at 100 W, followed by 4 min at 60 W. At the end of the procedure, the size of the ablated area was deemed adequate, with the formation of a ground-glass halo, measuring 3.5 × 2.1 × 2.0 cm, encompassing the entirety of the treated lesion (Figure 1B). The total treatment time was 30 min, and there were no complications.

### Case 2 - Liver (cholangiocarcinoma)

A 69-year-old male patient who undergone resection of an intrahepatic cholangiocarcinoma, measuring 10 cm, in segments VI and VII, in September 2016. During the oncological follow-up, he developed a new liver lesion, measuring 2.5 cm, identified in April 2019. The decision was made to treat the lesion with microwave ablation.

**Figure 2:**
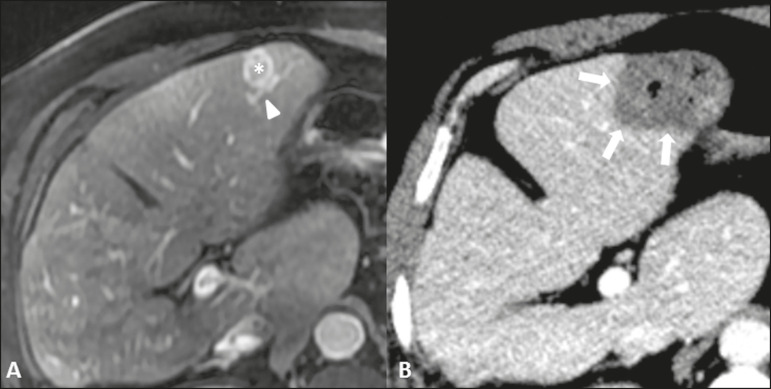
**A:** Magnetic resonance imaging scan of the upper abdomen, in the arterial phase, showing a lesion in liver segment III (asterisk). An arterial branch can be seen near the nodule (arrowhead). **B:** Follow-up computed tomography scan acquired after microwave ablation, showing a satisfactory ablation zone (arrows), with no heat-sink effect by the adjacent arterial branch (included in the ablation zone).

As in case 1, the procedure was performed under general anesthesia with the Solero MTA microwave tissue ablation system (AngioDynamics). In this case, the procedure was guided by computed tomography with a 40-channel scanner (Brilliance; Philips Medical Systems) and by ultrasound (Logiq E9; GE Healthcare, Chicago, IL, USA). The microwave antenna was introduced into the liver lesion by percutaneous insertion (Figure 2A), and microwave power was applied in a single cycle, at 60 W for 2 min. At the end of the procedure, the ablated area was deemed to be satisfactory in size, measuring 4.8 × 3.8 × 2.7 cm, completely encompassing the liver lesion (Figure 2B). The total treatment time was 30 min, and there were no complications.

## COMMENTARY

In our initial experience with microwave ablation, we found that this technique produces more extensive ablation zones in very short periods of time. In the lung, we achieved uniform ablation, without interference from high pulmonary impedance, despite the fact that the lesion treated was small; in the liver, microwave ablation produced a uniform, extensive area of ablation with only one access, without the need for overlapping applications.
